# A Physics-Conditioned Denoising Diffusion Probabilistic Model for Vibration-Based Wheel Flat Diagnosis Under Variable-Speed Conditions

**DOI:** 10.3390/s26216693

**Published:** 2026-10-22

**Authors:** Haoyi Yan, Songyuan Xu, Chuang Gao, Yang Chen, Bingrong Miao

**Affiliations:** State Key Laboratory of Rail Transit Vehicle System, Southwest Jiaotong University, Chengdu 610031, China; 2024212057@my.swjtu.edu.cn (H.Y.); 2024300681@my.swjtu.edu.cn (S.X.); chuangg@swjtu.edu.cn (C.G.); yang_chen@my.swjtu.edu.cn (Y.C.)

**Keywords:** wheel flat, variable-speed condition, physics-conditioned DDPM, data augmentation, fault diagnosis

## Abstract

Wheel flats deteriorate normal wheel-rail contact conditions and induce periodic impacts, thereby affecting the operational stability and reliability of railway vehicles. In practical engineering applications, the number of fault samples available for diagnosis is usually limited. Moreover, under variable-speed conditions, fault-related time-frequency characteristics in vehicle vibration responses vary significantly with operating speed, which further increases the difficulty of constructing labeled training datasets. To address these issues, a physics-conditioned denoising diffusion probabilistic model (DDPM)-based data augmentation method is proposed in this study. Impact occurrence timing and peak amplitude are extracted from vibration responses as response-derived physical conditions to characterize the temporal distribution of wheel-rail impacts and the variation in impact intensity. Subsequently, the physical conditions are transformed into two-dimensional conditional feature maps and introduced into the diffusion generation process, enabling the generation model to learn the evolution patterns of fault-related time-frequency characteristics caused by operating-condition variations. The proposed method is validated using a vehicle-track dynamic simulation dataset and a scaled vehicle-track test rig dataset. Compared with the original datasets, the proposed method improves the diagnosis accuracy from 87.8% to 94.4% on the simulation dataset and from 69.2% to 81.2% on the scaled vehicle-track test rig dataset. These results indicate that the proposed physics-conditioned DDPM can generate samples that better preserve impact-related time-frequency characteristics and improve wheel flat diagnosis performance under limited fault samples.

## 1. Introduction

The service condition of railway wheels directly affects the operational performance of railway vehicles. During long-term service, wheels are continuously subjected to complex wheel-rail interactions, which may lead to typical defects such as wheel flats [1,2,3]. Wheel flats further deteriorate the wheel-rail contact condition and induce abnormal vehicle vibrations, thereby accelerating the development of fatigue damage in the wheel-rail system [4]. Therefore, online monitoring and condition assessment of wheel flats are of great significance for achieving vehicle fault prediction and prognostics and health management [5,6,7].

In recent years, deep learning methods have been widely applied in the field of fault diagnosis [8,9,10]. These methods can automatically extract fault-related features from vibration signals or time-frequency representations, and the diagnostic performance can be improved through hierarchical feature learning [11,12,13,14]. For example, Ye et al. [15] proposed a hybrid method combining activated time-domain images (ATDI) and deep neural networks for wheel flat identification under different speed levels. Zhang et al. [16] proposed a multi-channel vibration feature fusion framework to capture complementary fault features. However, deep learning methods usually require a large number of labeled training samples. Due to complex operating conditions and other practical limitations, obtaining sufficient fault samples is often difficult. Therefore, achieving accurate fault diagnosis under limited training sample conditions remains an important challenge [17,18,19].

To address the problem of insufficient fault samples, data augmentation methods have gradually been applied for fault sample expansion [20,21,22]. Among these methods, the data-driven denoising diffusion probabilistic model (DDPM) generates samples through a progressive noise addition process and a reverse denoising process. It has the advantages of stable training and high generation quality, and has gradually been applied to fault sample generation tasks [23,24]. For example, Zhao et al. [25] utilized DDPM to generate two-dimensional time-frequency diagrams for fault diagnosis. Yu et al. [26] improved the noise prediction network to enhance the quality of generated samples.

However, existing DDPM-based augmentation methods mainly rely on learning the statistical distribution of training samples, while the physical mechanisms governing fault-response variations are rarely considered. Although conditional diffusion models can incorporate additional information, their conditions are generally based on labels or predefined attributes rather than fault-response-derived physical characteristics. For wheel flat faults, impact timing and response intensity vary with vehicle speed, resulting in speed-dependent changes in time-frequency characteristics. Therefore, introducing physically interpretable response information into diffusion generation provides a more direct constraint on fault-related feature evolution.

To address this issue, a physics-conditioned DDPM is proposed for wheel flat diagnosis under variable-speed conditions. Different from conventional DDPM and label-based conditional diffusion models, the proposed method introduces impact timing and peak amplitude extracted from vibration responses as response-derived physical conditions to guide sample generation. These conditions provide explicit constraints on impact distribution and intensity variation, enabling the generated samples to better preserve speed-dependent fault characteristics. The main contributions of this study are summarized as follows: (1) A physics-conditioned DDPM-based data augmentation framework is proposed for wheel flat sample generation. Different from existing conditional diffusion methods that mainly rely on class labels or predefined attributes, the proposed method introduces response-derived physical descriptors to guide the diffusion process. (2) Impact timing and peak amplitude extracted from vibration responses are transformed into two-dimensional condition maps and incorporated into the diffusion model, enabling the generated samples to preserve operating-condition-dependent fault-related characteristics. (3) The effectiveness of the proposed method is validated using both vehicle-track dynamic simulations and scaled vehicle-track experiments, demonstrating improved wheel flat diagnosis performance under limited fault samples.

The paper structure is described as follows: The theoretical background of DDPM, the physics-conditioned DDPM method, the Stockwell transform, and ResNet-18 are presented in Section 2. Section 3 describes the proposed data augmentation framework and the construction of physics-conditioned information. Section 4 reports the numerical and experimental validation results, including generated sample evaluation, comparison with different augmentation methods, feature distribution analysis, and test-rig validation. Finally, conclusions are drawn in Section 5.

## 2. Theoretical Background and Mathematical Model

### 2.1. Denoising Diffusion Probabilistic Model

**Figure 1:**
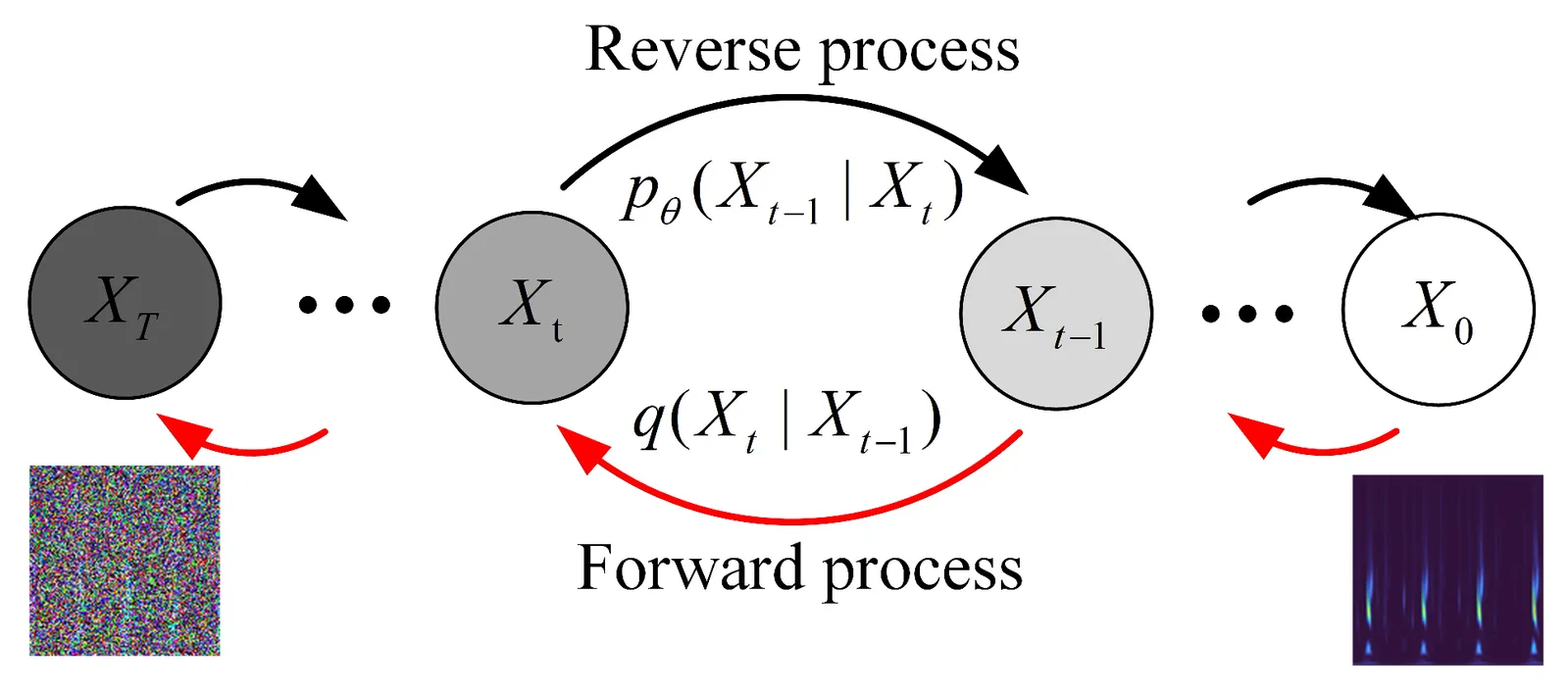
Schematic diagram of the DDPM.

DDPM can be regarded as a likelihood-based generation framework, where the data distribution is gradually transformed into a noise distribution and then recovered through a learned reverse sampling procedure [27]. As illustrated in Figure 1, Gaussian noise is progressively added to the original sample during the forward process, whereas the reverse process employs a neural network to estimate and remove the added noise step by step.

#### 2.1.1. Forward Process

The forward diffusion process is formulated as a Markov chain in which Gaussian noise is progressively added to the sample. The transition from $X_{t-1}$ to $X_{t}$ is defined as follows:

(1) $$q\left(X_{t}|X_{t-1}\right)=N\left(X_{t};\sqrt{1-\beta_{t}}X_{t-1},\beta_{t}I\right),$$

where $\beta_{t}$ denotes the predefined noise variance at diffusion step *t*.

The cumulative noise coefficient is described in Equations (2) and (3).

(2) $$\alpha_{t}=1-\beta_{t}$$

(3) $${\bar{\alpha }}_{t}=\prod_{s=1}^{t}\alpha_{s}$$

Based on this definition, the noisy sample at an arbitrary diffusion step can be directly obtained as follows:

(4) $$X_{t}=\sqrt{{\bar{\alpha }}_{t}}X_{0}+\sqrt{1-{\bar{\alpha }}_{t}}\varepsilon ,\;\varepsilon ∼N\left(0,I\right).$$

As *t* increases, ${\bar{\alpha }}_{t}$ gradually decreases and $X_{t}$ approaches Gaussian noise.

#### 2.1.2. Reverse Process

The reverse process progressively reconstructs the data from $X_{T}$ by learning the reverse transition distribution. This transition is parameterized by a neural network as follows:

(5) $$p_{\theta }\left(X_{t-1}|X_{t}\right)=N\left(X_{t-1};\mu_{\theta }\left(X_{t},t\right),\Sigma_{\theta }\left(X_{t},t\right)\right).$$

In the standard DDPM formulation, the posterior variance used in reverse sampling is given as follows:

(6) $${\tilde{\beta }}_{t}=\frac{1-{\bar{\alpha }}_{t-1}}{1-{\bar{\alpha }}_{t}}\beta_{t}.$$

#### 2.1.3. Objective Function

DDPM training can be derived from the variational lower bound of the data likelihood [27]. Under the standard noise-prediction parameterization, the optimization objective can be simplified to predicting the Gaussian noise added during the forward process:

(7) $$L_{\theta }=E_{X_{0},\varepsilon ,t}\left[{∥\varepsilon -\varepsilon_{\theta }\left(X_{t},t\right)∥}^{2}\right]$$

where $\varepsilon_{\theta }$ denotes the noise estimated by the neural network at diffusion step *t*. This standard objective provides the basis for extending the DDPM framework by incorporating response-derived physical conditions in the following section.

### 2.2. Physics-Conditioned DDPM

#### 2.2.1. Physics-Conditioned Representation and Diffusion Process

Conventional DDPM generates samples mainly by learning the implicit data distribution of the training data. However, under variable-speed conditions, wheel-flat-induced vibration responses exhibit operating-condition-dependent variations in both the temporal distribution and response intensity of impact-related characteristics. These variations are reflected in the response-derived physical information extracted from vibration signals rather than the speed value itself. If such physical response variations are not explicitly considered, some operating-condition-related fault characteristics may be weakened during the generation process. Therefore, a physics-conditioned DDPM is developed by introducing response-related physical information into the diffusion model. The response-derived physics-conditioned set is denoted as follows:

(8) $$C=\{c_{\mathrm{time}},c_{\mathrm{peak}}\}.$$

where $c_{\mathrm{time}}$ represents the temporal distribution of wheel flat impacts, and $c_{\mathrm{peak}}$ represents the corresponding impact intensity. Together, these two condition components provide complementary descriptions of the wheel flat vibration response from the temporal and amplitude perspectives.

Since the impact occurrence timing and amplitude are represented as one-dimensional temporal information, whereas the DDPM operates on two-dimensional time-frequency diagrams, the physical conditions are mapped into two-dimensional representations to achieve spatial alignment with the diffusion input. A Gaussian mapping function is employed for this purpose, as expressed in Equation (9).

(9) $$G_{i}\left(x\right)=\exp\left(-\frac{{\left(x-p_{i}\right)}^{2}}{2\sigma^{2}}\right)$$

where *x* denotes the time coordinate of the sampling point. The parameter $p_{i}$ represents the time-axis position of the *i*-th impact event, and σ is the Gaussian mapping width, which controls the spatial spread of the mapped impact information. In this study, σ is set to 1 s according to the ST window duration and the typical impact interval variation under the investigated speed range. This value provides sufficient smoothing of discrete impact locations while allowing neighboring impact responses to overlap and form a continuous temporal conditioning pattern. The Gaussian mapping retains the temporal location information of each impact event while providing a smooth local representation around the detected position, which facilitates its subsequent integration with the time-frequency features.

Based on the Gaussian mapping function, two one-dimensional physics-conditioned signals are first constructed by superimposing the contributions of all detected impact events. The temporal condition signal describes the distribution of impact locations, while the amplitude condition signal incorporates the corresponding impact amplitudes. These two conditioned signals are expressed as follows:

(10) $$c_{\mathrm{time}}\left(x\right)=\sum_{i}G_{i}\left(x\right)$$

(11) $$c_{\mathrm{peak}}\left(x\right)=\sum_{i}\left|A_{i}\right|\;G_{i}\left(x\right)$$

where $A_{i}$ denotes the peak amplitude of the *i*-th impact event. Here, $c_{\mathrm{time}}\left(x\right)$ encodes the temporal distribution of the detected impact events, whereas $c_{\mathrm{peak}}\left(x\right)$ further weights each event according to its corresponding response amplitude $|A_{i}|$. The two one-dimensional condition signals are subsequently expanded along the frequency dimension to obtain condition maps with the same spatial dimensions as the time-frequency samples. Unlike the normalized ST-based time-frequency representation, the peak-amplitude condition is directly derived from detected impact responses and retains the relative variation of impact intensity among different operating conditions. Therefore, the ST representation and peak-amplitude condition provide complementary information, where the former describes time-frequency evolution and the latter provides amplitude-related physical constraints.

The resulting two-dimensional condition maps are spatially aligned with the time-frequency samples and incorporated into the DDPM as conditioned information to guide the reverse denoising process.

During training, the two physics-conditioned maps are concatenated with the noisy time-frequency sample $X_{t}$ along the channel dimension and jointly fed into the noise-prediction network. Accordingly, the standard DDPM noise predictor $\varepsilon_{\theta }\left(X_{t},t\right)$ is extended to the condition-dependent form $\varepsilon_{\theta }\left(X_{t},t,C\right)$. The corresponding training objective is expressed as Equation (12), and the corresponding sampling procedure is detailed in Listing 1.

(12) $$L_{\theta }=E_{X_{0},\varepsilon ,t,C}\left[\|\varepsilon -\varepsilon_{\theta }\left(X_{t},t,C\right){\|}^{2}\right]$$

**Listing 1.** Physics-conditioned DDPM.Sample $X_{T}∼N\left(0,I\right)$ **for**
 t=T,…,1 **do**   **if**
 t>1 **then**     z∼N(0,I)   **else**     z=0   **end if**   $X_{t-1}=\frac{1}{\sqrt{\alpha_{t}}}\left[X_{t}-\frac{1-\alpha_{t}}{\sqrt{1-{\bar{\alpha }}_{t}}}\varepsilon_{\theta }\left(X_{t},t,C\right)\right]+\sqrt{{\tilde{\beta }}_{t}}\;z$ **end for** **return**
 $X_{0}$

Therefore, the introduced physical information does not alter the basic optimization formulation of DDPM; instead, it conditions the noise-prediction process so that the reverse diffusion is guided by the wheel flat response characteristics contained in *C*.

#### 2.2.2. U-Net with Time Embedding Fusion

To achieve effective fusion between time-frequency features and physics-conditioned information during the denoising process, a time-conditioned U-Net with an encoder-decoder architecture is adopted as the noise prediction network of the DDPM [28]. The network input is constructed by concatenating the noisy ST-based time-frequency images and two physics-conditioned maps along the channel dimension. The final input size is 5×125×128 (channels × height × width), including three RGB channels of the ST-based time-frequency image and two channels of physics-conditioned information. Through channel-wise concatenation, both time-frequency features and physical conditions can be provided to the U-Net at the input stage. To further enhance the interaction between time-frequency features and physics-conditioned information, a cross-attention mechanism is employed as an auxiliary feature fusion operation during the denoising process. In this study, the cross-attention module is used to improve the interaction between intermediate features and conditional information, while the main contribution lies in introducing response-derived physical conditions into the diffusion generation process.

**Figure 2:**
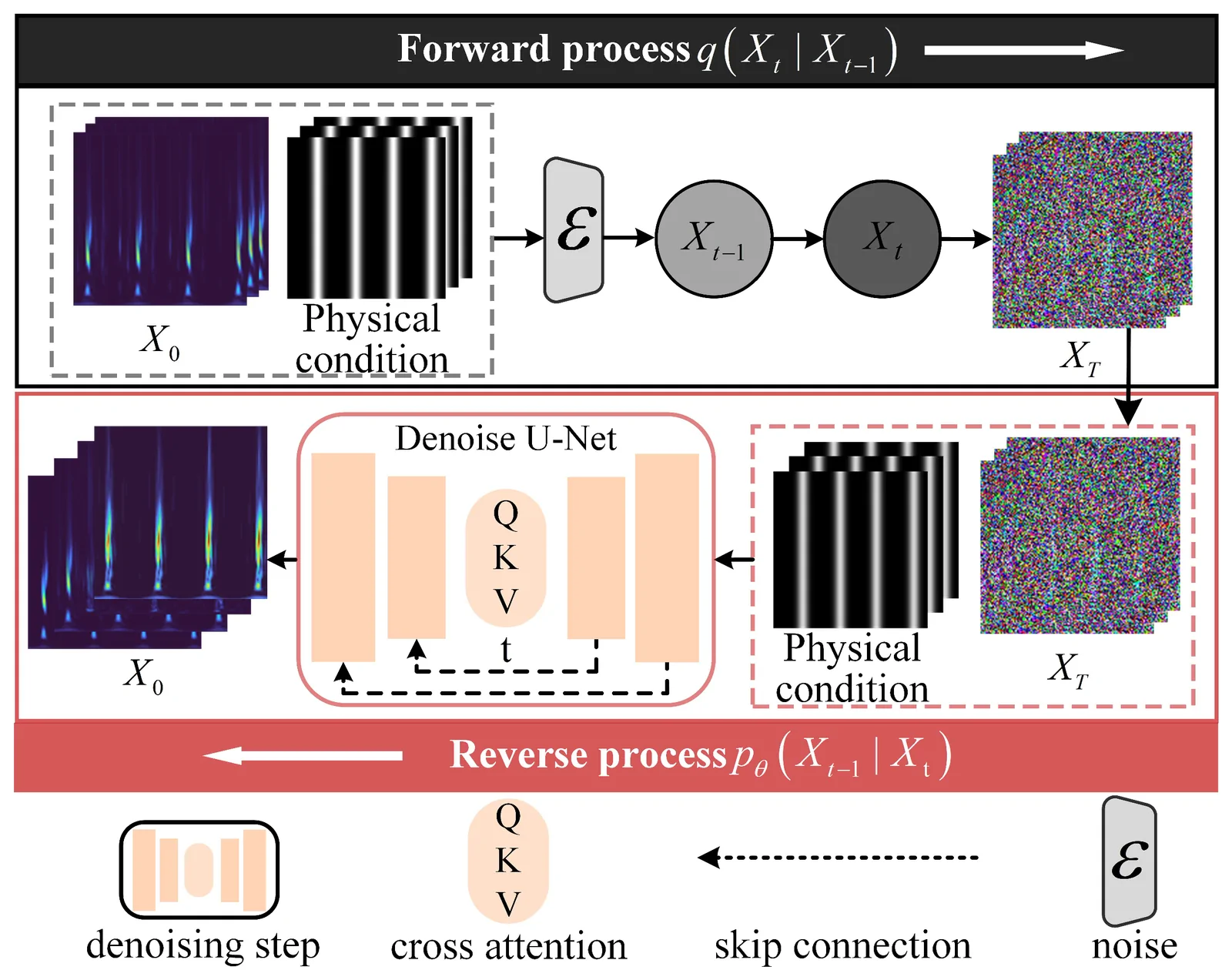
Architecture of the proposed physics-conditioned DDPM.

To incorporate the diffusion-step information, sinusoidal positional encoding is applied to the diffusion timestep *t*. The time-step embedding is then obtained through two fully connected layers. Subsequently, the time embedding is projected to the corresponding feature channels and introduced into the bottleneck and decoder stages of the U-Net. In this way, different denoising stages during the reverse diffusion process can be distinguished by the network. The overall architecture of the proposed physics-conditioned DDPM is illustrated in Figure 2.

### 2.3. Stockwell Transform

Under variable-speed conditions, wheel-flat-induced axle-box vibration responses exhibit significant non-stationary characteristics. With changes in vehicle speed, the impact interval and spectral-energy distribution vary over time. Therefore, fault-related characteristics are simultaneously reflected in both temporal and frequency domains. Time-domain signals can preserve transient impact responses but cannot directly describe spectral evolution, whereas frequency-domain analysis lacks temporal localization capability. Consequently, a time-frequency representation is required to characterize the evolution of wheel-flat-induced impact responses under variable-speed conditions.

The Stockwell transform (ST) combines the advantages of the short-time Fourier transform and the wavelet transform by employing a frequency-dependent Gaussian window, which provides adaptive time-frequency resolution. Therefore, it is suitable for analyzing vibration responses containing localized transient impacts and speed-dependent spectral variations [29]. The ST of a vibration signal h(t) is defined as:

(13) $$s\left(\tau ,f\right)=\int_{-\infty }^{+\infty }h\left(t\right)\cdot \omega \left(t-\tau ,f\right)\cdot e^{-i2\pi ft}\;dt$$

(14) $$\omega \left(t-\tau ,f\right)=\frac{\left|f\right|}{\sqrt{2\pi }}e^{-\frac{{\left(t-\tau \right)}^{2}f^{2}}{2}}$$

where τ denotes the time-localization parameter, and *f* denotes the frequency variable.

**Figure 3:**
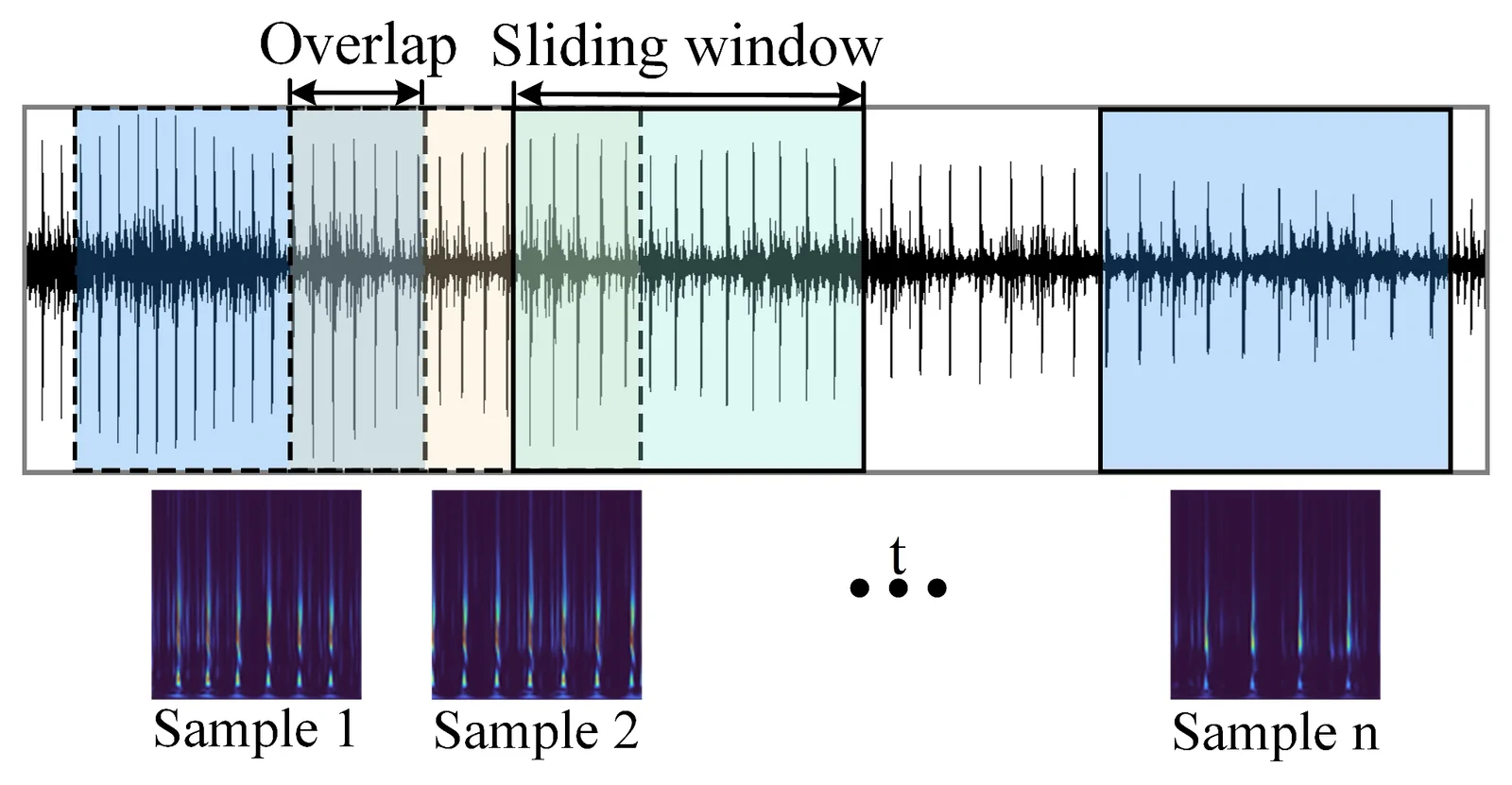
Sliding window algorithm for ST sampling.

In this study, the ST is applied to one-dimensional axle-box vibration signals to obtain two-dimensional time-frequency representations. The obtained time-frequency representations are encoded as RGB images to provide three-channel inputs for the diffusion model. These representations preserve the temporal locations of wheel flat-induced impacts and characterize the evolution of spectral-energy distributions under different operating conditions. Therefore, the ST-based time-frequency diagrams provide an appropriate representation domain for the proposed physics-conditioned DDPM, where the generated samples can be guided by response-derived physical conditions to maintain consistent fault-related characteristics. The corresponding signal segmentation and ST-based representation procedure are illustrated in Figure 3.

### 2.4. Residual Neural Network

In this study, ResNet-18 is adopted as the classifier for wheel flat diagnosis to evaluate the effectiveness of different data augmentation methods. Owing to its residual learning structure, ResNet-18 can extract hierarchical features from two-dimensional time-frequency representations. The original fully connected layer is replaced according to the number of fault categories to achieve multi-class fault identification. The network is trained using the cross-entropy loss function, and the Adam optimizer is employed for parameter updating. Finally, the diagnostic performance is evaluated using the test accuracy and confusion matrix.

## 3. Data Augmentation Method

### 3.1. Physics-Conditioned Data Augmentation Framework

**Figure 4:**
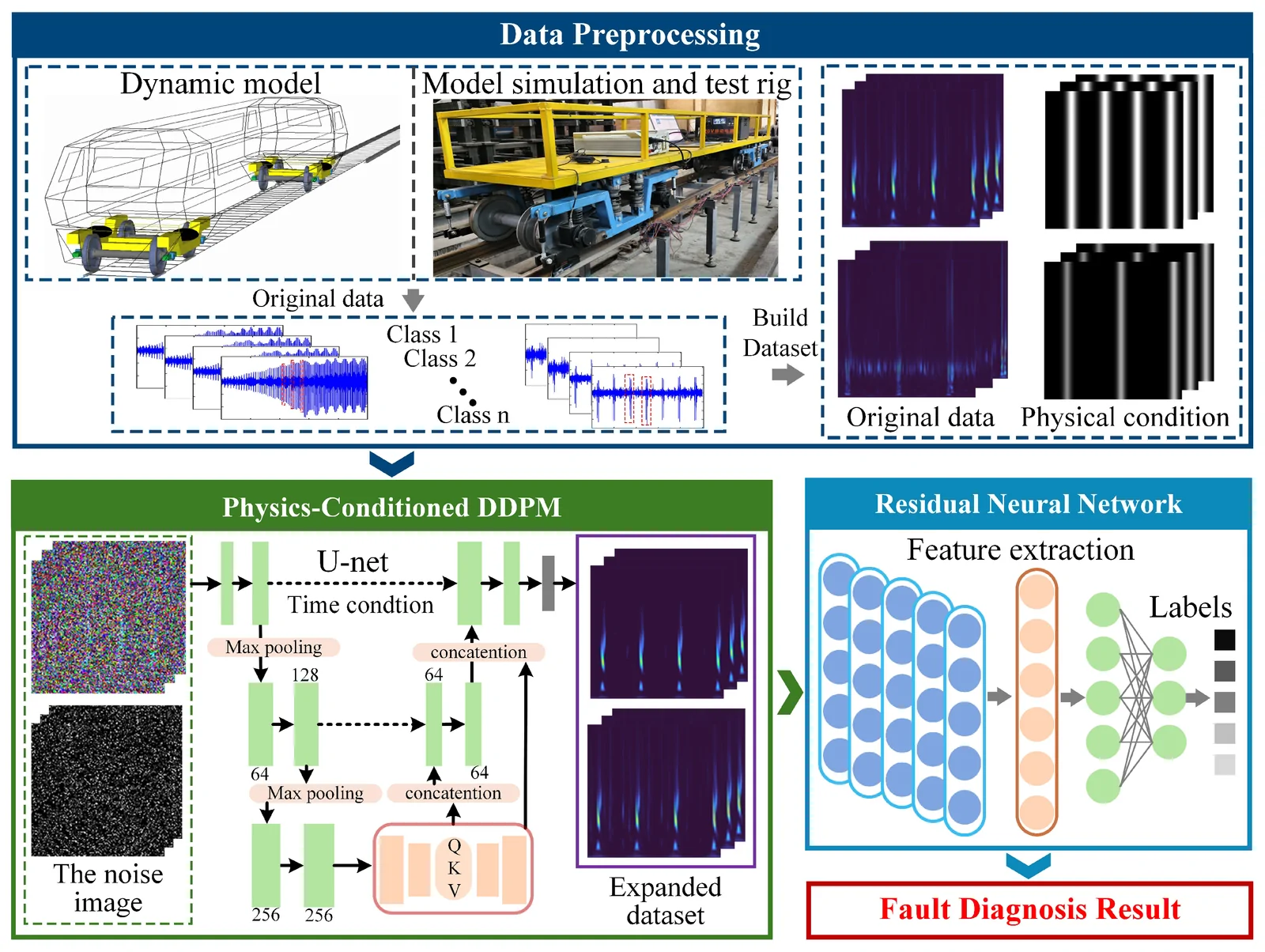
Flowchart for wheel flat diagnosis with data augmentation.

To improve the consistency of generated fault responses under variable-speed conditions, a physics-conditioned DDPM-based data augmentation framework is proposed for wheel flat diagnosis, as shown in Figure 4.

First, a vehicle-track rigid-flexible coupled dynamics model considering wheel flats is established, and axle-box vibration responses under different variable-speed conditions are obtained. Subsequently, the non-stationary vibration signals are transformed into two-dimensional time-frequency representations using the Stockwell transform (ST). Meanwhile, impact occurrence timing and peak amplitude are extracted from the vibration responses as physics-conditioned information. Vehicle speed is not directly used as the generation condition. Instead, speed-related variations are represented by the extracted impact timing and peak amplitude, which describe the corresponding changes in impact interval and intensity.

Subsequently, the time-frequency diagrams and physics-conditioned information are jointly introduced into the DDPM to construct a condition-guided sample generation framework. To avoid data leakage during the augmentation process, the physics-conditioned DDPM is trained only using samples from the training set. The physical conditions used for generation are extracted exclusively from the corresponding training samples, while validation and test samples are never involved in model training or condition construction. All generated samples are subsequently combined with the original training samples for classifier training. A physics-conditioned DDPM is trained using all fault categories, while class-specific physical conditions extracted from the corresponding training samples are used to guide sample generation. The generated samples are assigned the same diagnostic labels as their corresponding source samples for subsequent classifier training. The physical conditions constrain the generation process to represent the time-frequency structures associated with operating-condition variations. Therefore, the consistency of fault-related time-frequency characteristics in the generated samples can be improved.

Then, the generated samples are integrated with the original training samples to construct an augmented dataset. ResNet-18 is adopted as the classifier for wheel flat diagnosis. To guarantee a fair comparison, the same training parameters and classifier configurations are maintained across different data augmentation methods. The performance of the proposed method is assessed based on generated sample consistency and diagnostic performance.

Finally, experimental validation is conducted using a scaled vehicle-track test rig. The effectiveness of the proposed augmentation method under measured vibration conditions is further evaluated.

### 3.2. Construction of Physics-Conditioned Information

Wheel flats induce periodic abnormal wheel-rail contacts, which generate transient impacts in axle-box vibration signals. Under variable-speed conditions, the impact interval varies with vehicle speed through the change in wheel rotational speed, while the impact amplitude is also affected by operating conditions. Therefore, impact events are extracted from vibration responses to construct the physics-conditioned information. A sliding-window-based dynamic threshold is employed to identify impact events:

(15) $$\theta \left(t\right)=\lambda M_{w}\;\left(|x(t)|\right)$$

where $M_{w}$ denotes the moving-average operator with a window length of 1 s and a moving step of 0.5 s. The window length and coefficient λ are empirically determined according to the vibration characteristics under different speed conditions, with λ set within 6–10 to achieve adaptive impact detection. The minimum admissible interval between adjacent impacts is further estimated based on the maximum vehicle speed to avoid repeated detection, as expressed below:

(16) $$T_{\min}\approx \frac{\pi D}{v_{\max}}$$

where *D* represents the wheel diameter and $v_{\max}$ represents the maximum vehicle speed considered in this study. The calculated interval corresponds to the shortest possible inter-impact interval within the investigated speed range and is used as a fixed minimum separation constraint to avoid repeated detection of the same impact event. Since $T_{\min}$ is determined under the maximum-speed condition, the actual impact interval under lower-speed or deceleration conditions remains larger than this threshold under the investigated operating conditions. Therefore, valid impact events during deceleration can still satisfy the interval criterion and will not be removed.

When $|x\left(t_{i}\right)|\;>\theta \left(t_{i}\right)$ and $t_{i}-t_{i-1}>T_{\min}$ are satisfied, an effective impact is identified. The impact peak is defined as the maximum absolute acceleration within each detected impact region, ensuring that the extracted amplitude represents the maximum wheel-rail impact response. The extracted peak amplitude is used to construct $c_{\mathrm{peak}}$, while the corresponding occurrence time is used to construct $c_{\mathrm{time}}$.

Both physical conditions are directly extracted from vibration responses without requiring class labels. Impact timing and peak amplitude are selected to characterize the occurrence and intensity of wheel-flat-induced impacts. Although these response-derived descriptors may reflect the combined effects of speed, defect geometry, axle load, track stiffness, and sensor location, they do not fully represent the underlying vehicle–track dynamics.

## 4. Numerical Simulation and Experimental Validation

### 4.1. Vehicle-Track Rigid-Flexible Coupled Dynamics Modeling

**Figure 5:**
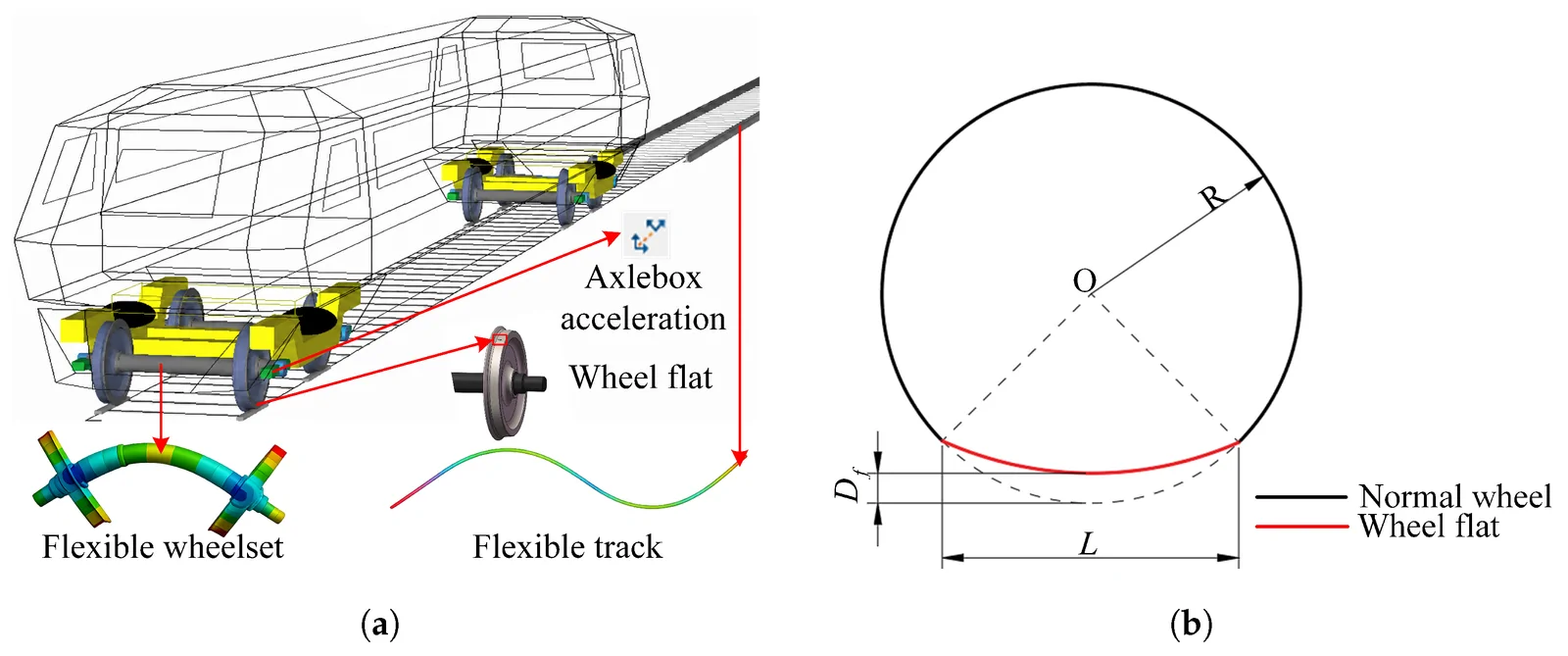
(**a**) vehicle-track rigid-flexible coupled dynamics model, (**b**) schematic of a wheel flat.

To make the dynamic simulation model better represent actual operating conditions and obtain accurate vehicle vibration responses and wheel-rail excitation characteristics, a vehicle-track rigid-flexible coupled dynamics model of a certain type of metro vehicle in China is established, as shown in Figure 5. In the model, the wheels and rails are considered as flexible bodies. The car body and bogie are connected through the secondary suspension system, while the bogie and wheelset are connected through the primary suspension system. All suspension systems are simplified as linear spring-damper elements.

Based on the vehicle–track coupled dynamics theory [4], the geometric irregularity of rounded wheel flats is described as a displacement input function:

(17) $$\eta =\frac{1}{2}D_{f}\left[1-\cos(2\pi x/L)\right]\left(0\leq x\leq L\right)$$

(18) $$D_{f}=\frac{L^{2}}{16R}$$

where η represents the irregularity of the rounded wheel flat, *L* represents the wheel flat length, $D_{f}$ represents the effective wheel flat depth, *x* represents the displacement along the wheel flat length, and *R* represents the wheel radius. The schematic diagram of the wheel flat structure is shown in Figure 5b. When a wheel flat defect exists, the wheel rotational frequency can be calculated as follows:

(19) $$f\left(t\right)=\frac{v(t)}{2\pi R}$$

where v(t) represents the instantaneous vehicle speed in m/s, and *R* represents the wheel radius. In the simulation, predefined acceleration and deceleration trajectories are adopted to generate variable-speed vibration responses. Vehicle speed is not directly used as the generation condition; instead, speed-related variations are represented by the response-derived physical conditions.

### 4.2. Simulation Response Analysis and Physics-Conditioned Information

**Figure 6:**
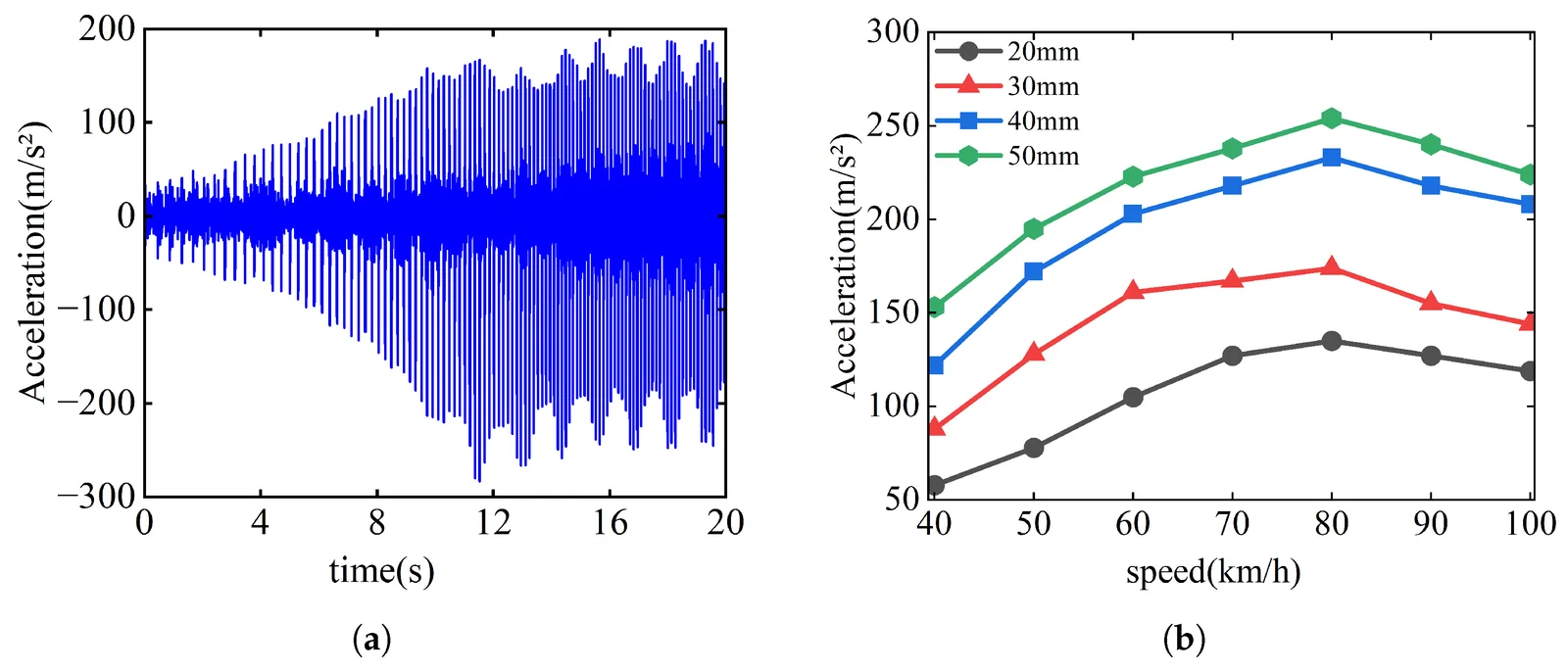
Axle-box vibration responses: (**a**) acceleration signal induced by a 40 mm wheel flat, (**b**) response variations under different wheel flat lengths and speeds.

To obtain wheel flat response data and construct the physics-conditioned information set, the vehicle vibration response signals induced by wheel flat defects are calculated and analyzed based on the vehicle-track rigid-flexible coupled dynamics model [30]. Since both the primary and secondary suspension systems attenuate the vibration responses caused by abnormal wheel-rail contact, the axle-box is selected as the signal measurement point [31,32,33]. The simulated time-varying vertical acceleration signals of the axle-box are shown in Figure 6, where the sampling frequency is 10 kHz. The related parameters of the dynamic model are listed in Table 1.

**Figure 7:**
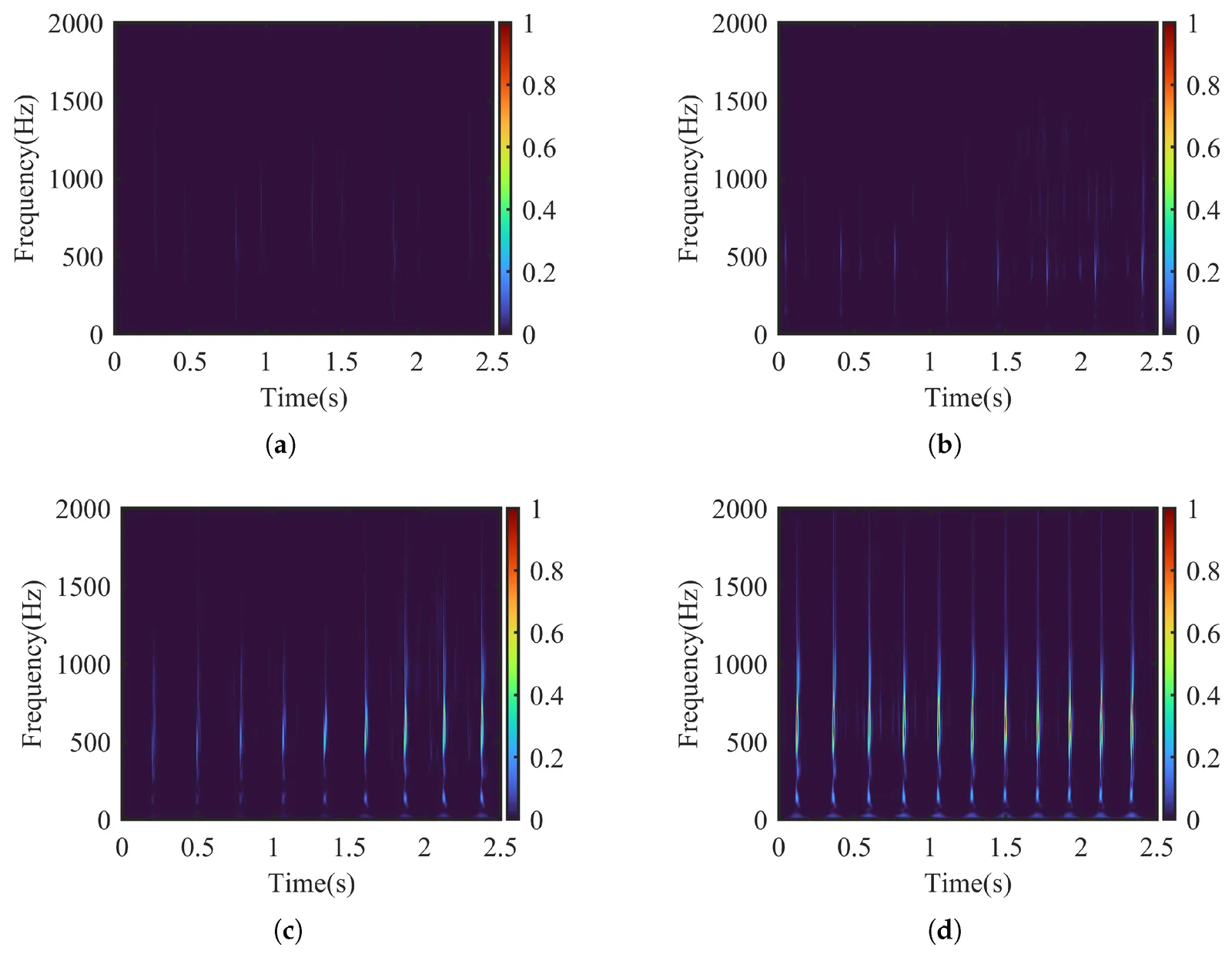
Time-frequency representations under variable-speed conditions: (**a**) no damage, (**b**) low speed, (**c**) mid speed, (**d**) high speed.

The variation of axle-box acceleration responses under different defect severities and vehicle speeds is shown in Figure 6b. The acceleration response is jointly affected by wheel flat length and vehicle speed. The peak value increases with increasing wheel flat length, while a nonlinear variation is observed with increasing vehicle speed. This indicates that the impact response amplitude is affected by both defect severity and operating conditions rather than vehicle speed alone. Therefore, the response peak can provide a direct description of wheel-flat-induced impact intensity. To further investigate the time-varying characteristics of fault responses under variable-speed conditions, the Stockwell transform (ST) window length is set to 2.5 s. Each ST representation is normalized to improve input consistency while preserving the relative time-frequency characteristics of wheel-flat impacts. Although normalization reduces amplitude differences in ST images, the peak-amplitude-based physics condition is extracted from the original vibration responses to retain impact intensity information. Therefore, the two inputs provide complementary time-frequency and amplitude-related physical information. The corresponding time-frequency diagrams are shown in Figure 7.

**Figure 8:**
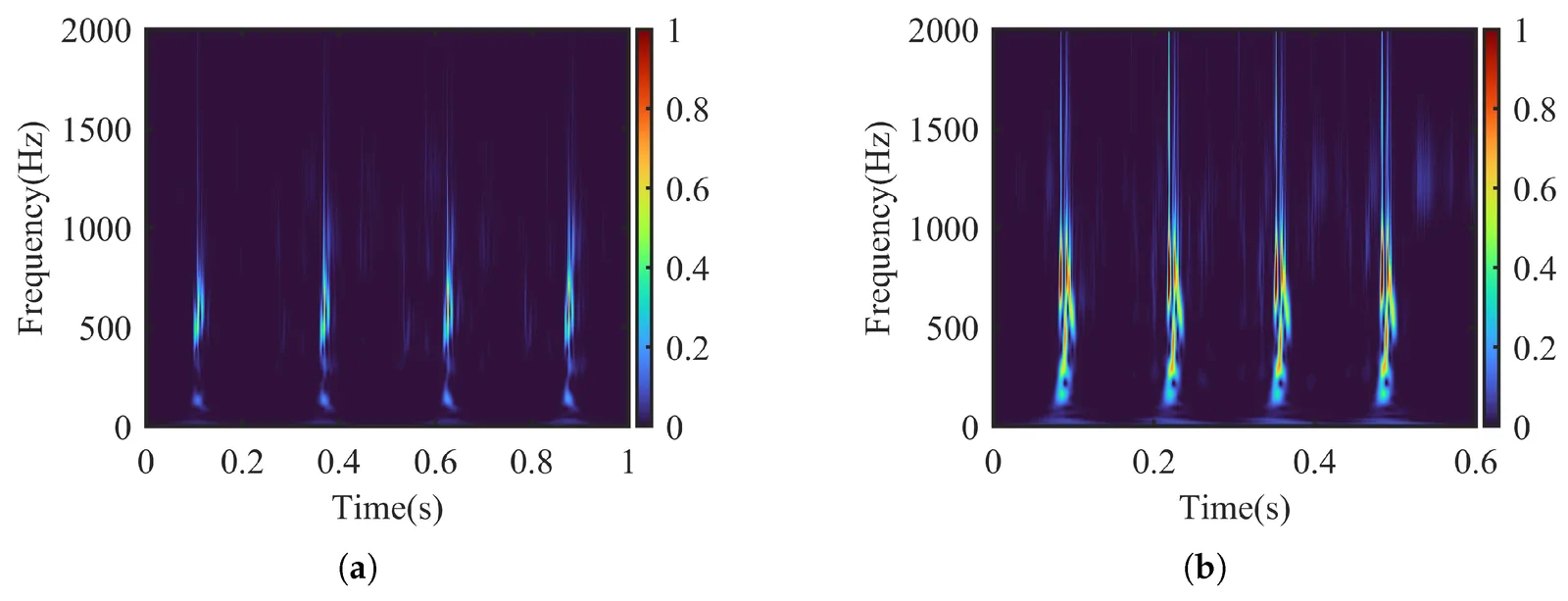
Time-frequency diagrams of the same wheel flat defect at different speeds after sliding-window segmentation: (**a**) low speed and (**b**) high speed.

As shown in Figure 7, clear periodic vertical stripe structures can be observed in the time-frequency diagrams. These stripe structures correspond to the periodic wheel-rail impact process induced by wheel flats. The stripe interval and local energy intensity vary with vehicle speed. With increasing vehicle speed, the stripe interval gradually decreases, which is consistent with the shortened wheel rotational period and reduced interval between adjacent wheel flat impacts under high-speed conditions. Meanwhile, changes in local energy intensity are observed under varying speed conditions. Such results indicate that the time-frequency representation can effectively describe the temporal distribution and energy variation of wheel flat impacts. To further investigate the influence of vehicle speed on the time-frequency evolution of wheel flat responses, the window range is adjusted so that each window contains four consecutive impact segments. The corresponding time-frequency diagrams are shown in Figure 8.

As shown in Figure 8, different time-frequency distribution characteristics are observed for the same wheel flat defect under different speed conditions. With increasing vehicle speed, the interval between adjacent impacts decreases, while the frequency-energy distribution gradually shifts toward higher frequencies with a broader bandwidth. These variations indicate that the fault-related time-frequency characteristics are strongly associated with operating conditions. Although the above time-frequency variations can describe the evolution of wheel-flat responses, they are coupled response characteristics affected by multiple dynamic factors and cannot be directly used as physical conditions. In contrast, impact occurrence timing and peak amplitude can be directly extracted from vibration responses and have clear physical meanings. Therefore, impact occurrence timing and peak amplitude are selected as physics-conditioned variables to guide the DDPM generation process.

### 4.3. Simulation Dataset Construction and Augmentation Evaluation

After obtaining the original vibration signals through dynamic simulation, five simulation runs were performed with randomly assigned vehicle positions to account for variations in the simulated vibration responses. The resulting continuous vibration signals were divided into training, validation, and test subsets at a ratio of 3:1:1 before sliding-window segmentation. All samples extracted from the same simulation run were assigned to the same subset, ensuring that samples originating from the same run did not appear in different subsets. Sliding-window segmentation was then performed independently within each subset, resulting in an original dataset containing 2500 samples. Different data augmentation methods were subsequently applied only to the training set after data splitting. The validation and test sets remained unchanged during data augmentation and classifier training to prevent direct data leakage. However, the augmentation ratio was selected based on test-set performance, as discussed below.

#### 4.3.1. Generated Sample Quality Analysis

**Figure 9:**
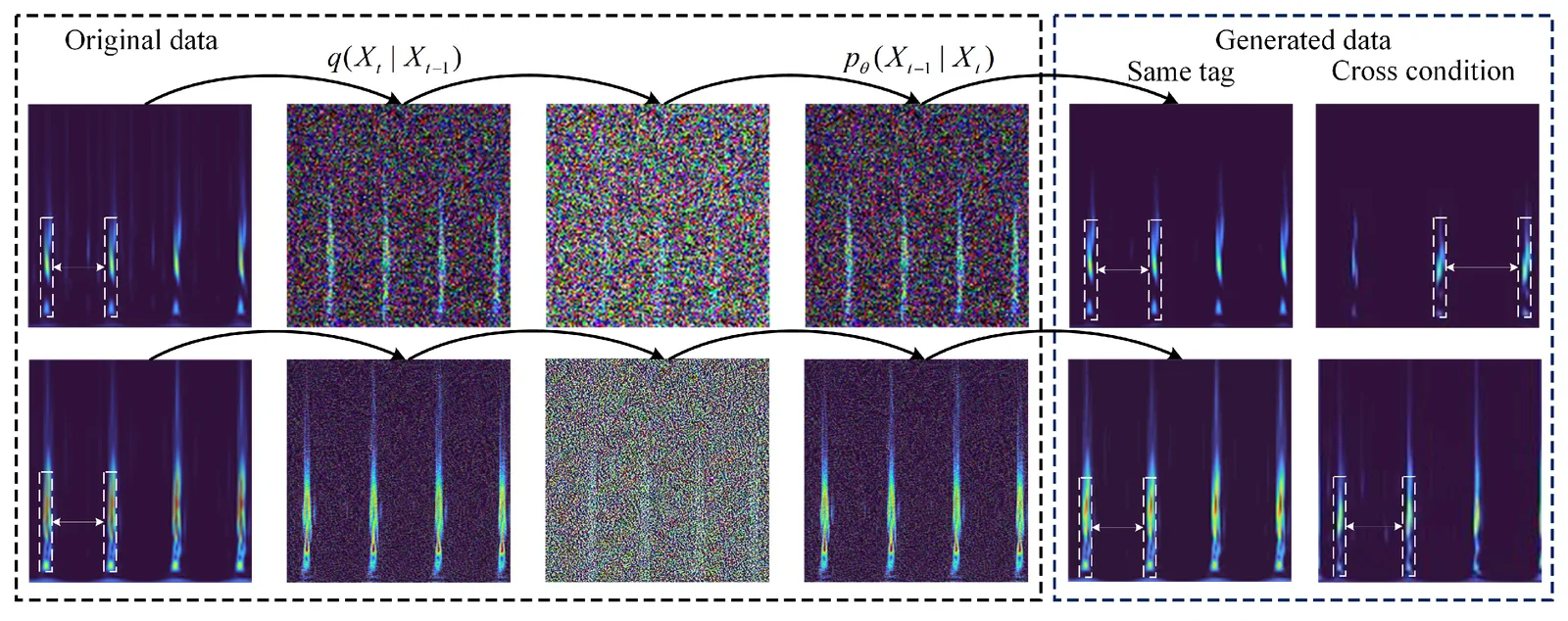
Comparison between original and generated time-frequency samples.

During training, the number of epochs is set to 10,000, the number of diffusion time steps is set to 400, the batch size is set to 128, and the learning rate is set to $1\times 10^{-4}$. The original samples and samples generated by the physics-conditioned DDPM are shown in Figure 9.

It can be observed that the generated samples exhibit clear and stable impact stripe structures, showing high consistency with the original samples, with only slight differences in edge regions and background structures. To further verify the modeling capability of the proposed method, additional conditional inputs representing different operating conditions are selected for sample generation, and the generated results are compared. This analysis investigates how different condition inputs affect the time-frequency structures of generated samples, and does not alter the experimental settings where variable-speed conditions are the primary research focus. Altering the physical conditioning inputs induces shifts in the temporal locations of impact-related stripes and the local energy distribution of synthetic samples. These trends are consistent with the physical roles of impact timing and amplitude, indicating that the introduced conditioning information can effectively guide the reconstruction of operating-condition-dependent time-frequency features.

**Figure 10:**
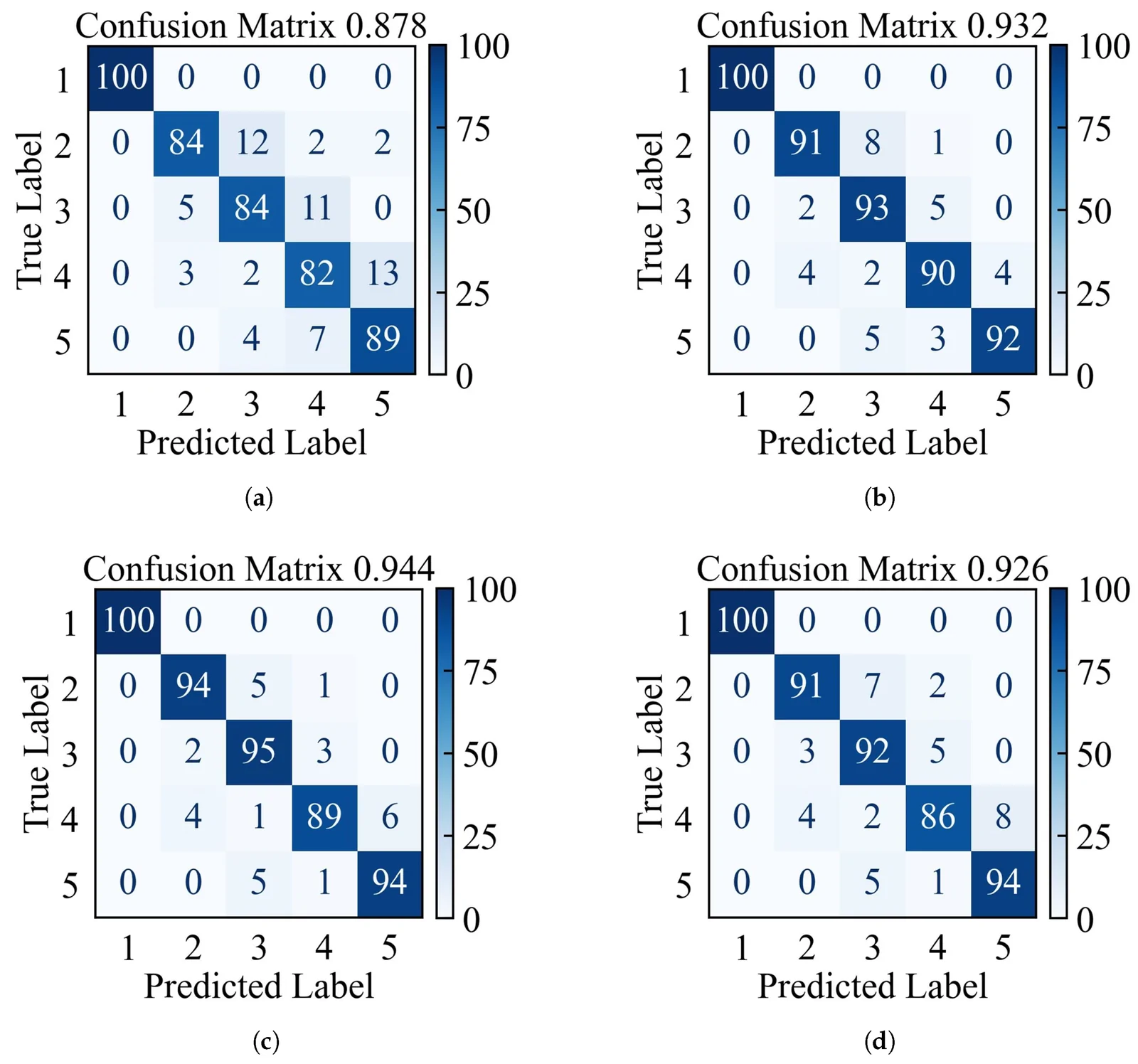
Confusion matrices on different datasets: (**a**) original dataset, (**b**) 750 generated samples, (**c**) 1500 generated samples, and (**d**) 3000 generated samples.

To further verify the feasibility and effectiveness of the proposed approach for fault classification, three augmentation settings are considered. Specifically, 750, 1500, and 3000 samples are generated, corresponding to augmentation ratios of 0.5, 1, and 2 relative to the original training set. An equal number of samples is generated for each class. The class-specific physical conditions are randomly selected from the corresponding training samples, and the generated samples are assigned the same labels as the selected source samples. For each class, the physical conditions extracted from the corresponding real training samples are used to guide sample generation. The generated samples inherit the labels of the source training samples, while no explicit class labels are introduced into the diffusion process. Class 1 represents the no-damage condition, while classes 2, 3, 4, and 5 correspond to wheel flat lengths of 20, 30, 40, and 50 mm. ResNet-18 is trained for 300 epochs with a batch size of 32, and the Adam optimizer is adopted for model training. The accuracies obtained using the original and augmented datasets are listed in Table 2, and the corresponding confusion matrices are presented in Figure 10.

As shown in Table 2, the highest classification accuracy of 94.4% is achieved at an augmentation ratio of 1, corresponding to an improvement of 6.6 percentage points over the original dataset. When the augmentation ratio is increased to 2, the accuracy decreases to 92.6%. These results indicate that the introduction of physically consistent generated samples can effectively improve diagnosis performance under limited training samples. However, excessive augmentation may introduce distribution bias when the generated samples exceed the effective diversity supported by the original data distribution. Considering both classification performance and training cost, the augmentation setting with a ratio of 2 is not used in the subsequent experiments.

#### 4.3.2. Comparison with Different Generative Models

**Figure 11:**
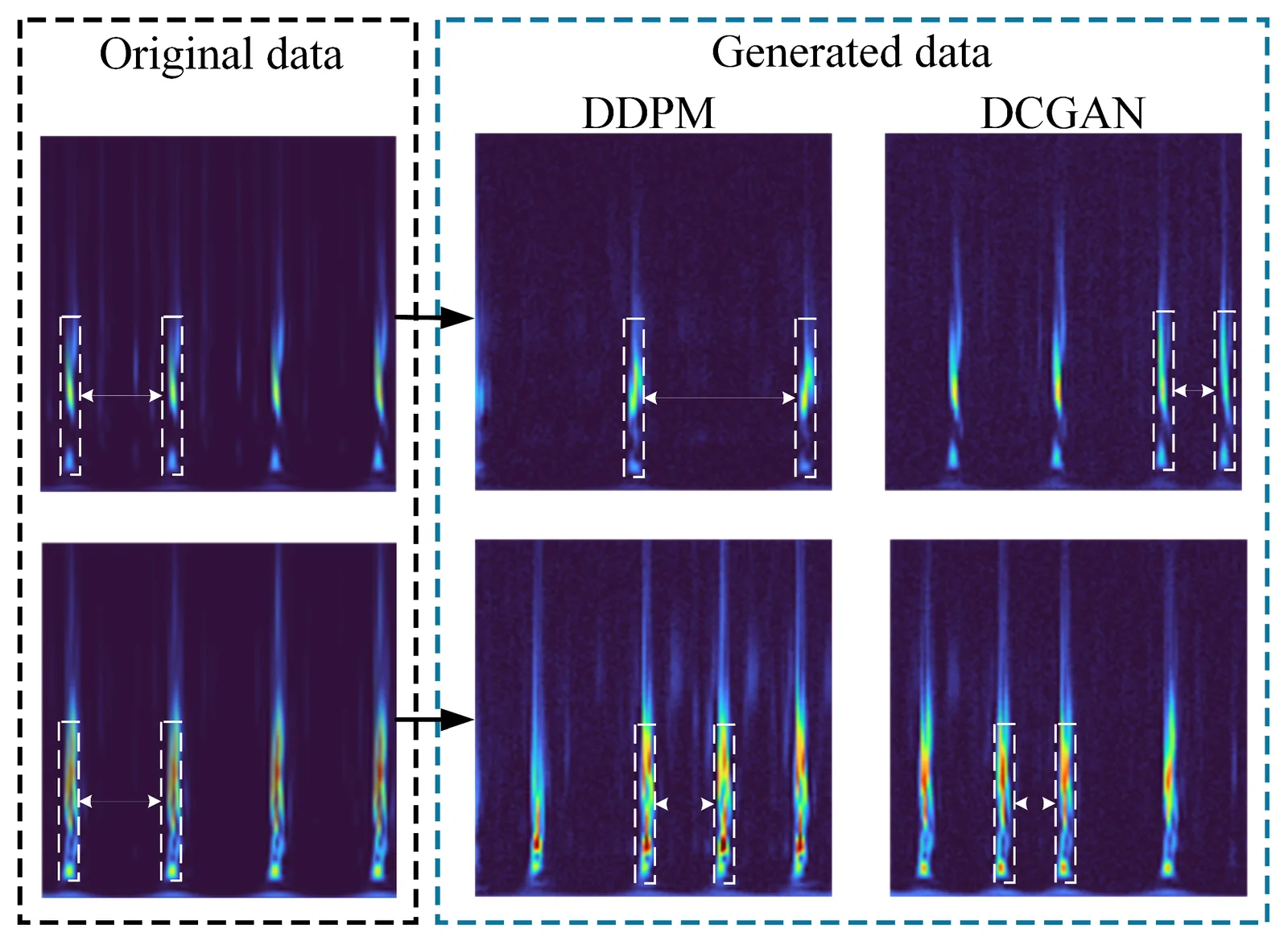
Original samples and samples generated by DDPM and DCGAN.

With the training parameters kept unchanged, samples are generated using DDPM and DCGAN, as shown in Figure 11. It can be observed that the basic vertical stripe structures are retained in the samples generated by DDPM. However, differences in the positions, intervals, and continuity of some impact stripes are still observed when compared with the original samples. Some impact-related characteristics are also preserved in the samples generated by DCGAN. However, the local texture details are relatively poor, and a certain degree of feature repetition is observed, indicating reduced sample diversity.

**Figure 12:**
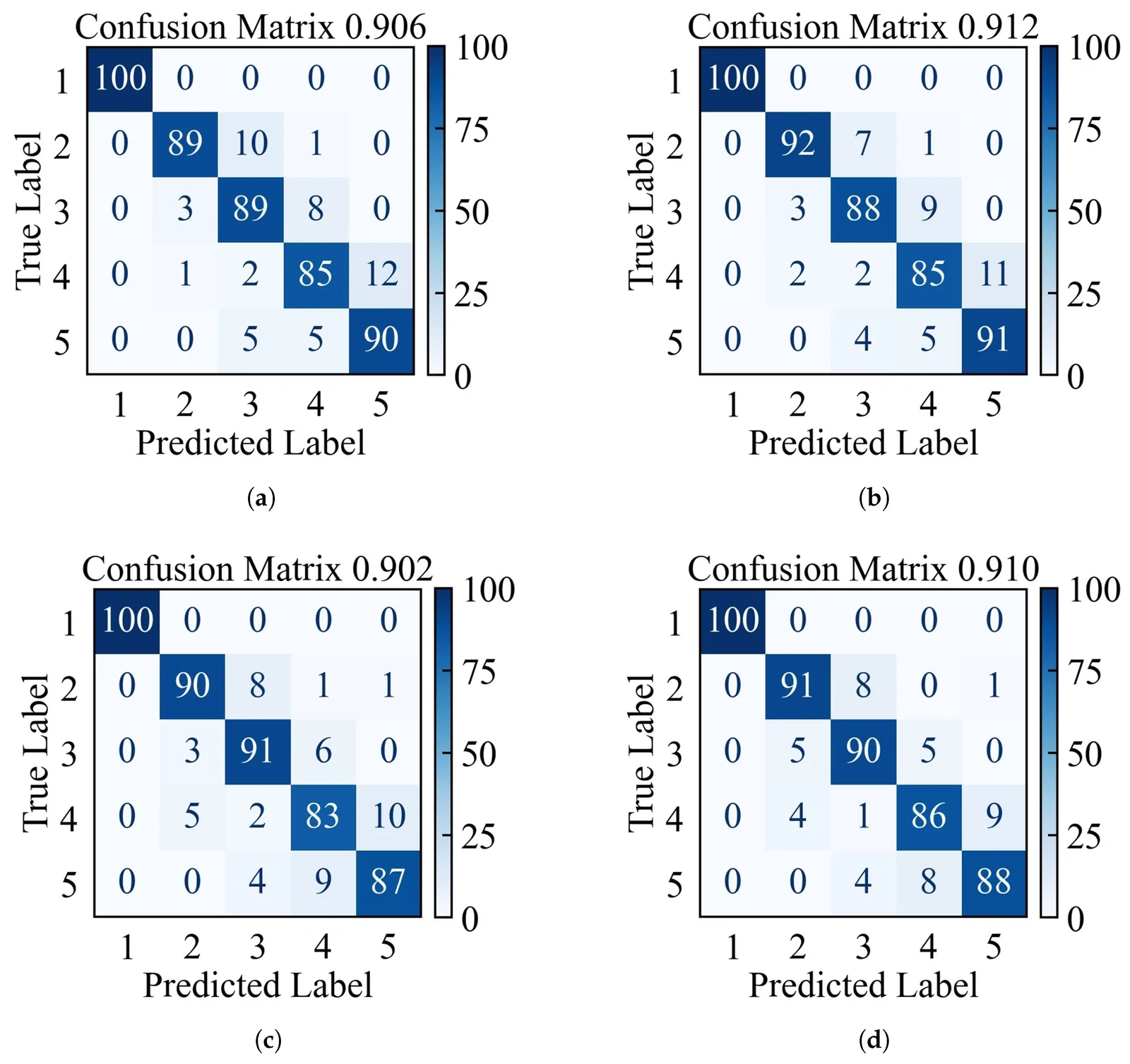
Confusion matrices of different models: (**a**) DCGAN with 750 generated samples, (**b**) DCGAN with 1500 generated samples, (**c**) DDPM with 750 generated samples, and (**d**) DDPM with 1500 generated samples.

With the validation and test sets kept unchanged, the original training set is augmented using samples generated by DDPM and DCGAN under the same augmentation settings described above. The diagnosis accuracies obtained from the augmented datasets are listed in Table 3, and the confusion matrices of DCGAN and DDPM are shown in Figure 12.

As shown in Table 3, data augmentation using DDPM and DCGAN can alleviate the problem of insufficient training samples to some extent. When 1500 samples are generated, the classification accuracy is 91.0% for DCGAN and 91.2% for DDPM. In contrast, a higher accuracy of 94.4% is achieved by the physics-conditioned DDPM. This difference may be partly attributed to the absence of response-derived physical information in DDPM and DCGAN. Without the constraints of impact timing and impact amplitude, sample generation mainly relies on the implicit data distribution of the training data. Therefore, the generated samples may not fully preserve the speed-dependent temporal distribution and energy characteristics of wheel flat impacts. In contrast, the proposed method introduces physics-conditioned information into the diffusion process, enabling the generated samples to retain more consistent fault-related time-frequency characteristics. In addition, adjacent defect classes share relatively similar features, while incomplete characteristics in the generated samples may introduce a certain degree of distribution bias. This may also partly explain why the recognition accuracy does not vary monotonically with defect severity. Therefore, an augmentation ratio of 1 is adopted in the subsequent comparison and experimental validation.

#### 4.3.3. Ablation Study and Robustness Analysis

To further investigate the contribution of different physics-conditioned components, an ablation study is conducted by removing impact timing and peak amplitude conditions separately. Since the cross-attention module only serves as a feature fusion operation and does not introduce additional physical constraints, the ablation study focuses on the response-derived physical conditions, including impact timing and peak amplitude, which constitute the main contribution of this study.

For the ablation experiments, one physical condition component is removed while the remaining generation process and diffusion parameters are kept unchanged. ResNet-18 trained only on real training samples is adopted as a fixed feature extractor. Feature vectors of generated and real samples are extracted from the penultimate layer of the network, allowing different ablation settings to be evaluated in the same feature space. The feature distribution distance is defined as follows:

(20) $$D_{\mathrm{feat}}={∥\mu_{r}-\mu_{g}∥}_{2}^{2}+\mathrm{Tr}\left(\Sigma_{r}+\Sigma_{g}-2{\left(\Sigma_{r}\Sigma_{g}\right)}^{1/2}\right)$$

where $\mu_{r}$ denotes the mean feature vector of the real training samples, while $\mu_{g}$ denotes that of the generated samples. Similarly, $\Sigma_{r}$ and $\Sigma_{g}$ represent the covariance matrices of the real and generated samples. $D_{\mathrm{feat}}$ is calculated separately for each fault category and then averaged over all categories. A lower $D_{\mathrm{feat}}$ indicates that the generated samples are closer to the real samples in the learned feature space. Since the feature extractor is trained using real samples, this metric mainly reflects feature-level distribution consistency and cannot independently evaluate generation diversity or exclude potential sample similarity. Therefore, $D_{\mathrm{feat}}$ is only used as a complementary metric in the ablation study to analyze the contribution of different physics-conditioned components, rather than as the sole criterion for evaluating generation quality.

To evaluate the sensitivity of the proposed method to uncertainties in physical condition estimation, a robustness experiment is conducted by introducing a 10% random perturbation into the extracted impact timing and peak-amplitude descriptors before Gaussian mapping. The perturbed descriptors are subsequently transformed into two-dimensional condition maps and used for sample generation, while the diffusion model parameters remain unchanged. This experiment focuses on the influence of physical condition estimation errors on sample generation, rather than the effect of measurement noise on the complete impact detection process.

For each ablation and robustness setting, 1500 samples are generated and evaluated using the same feature extractor. All models are trained under the same diffusion parameters, and only the physics-conditioned information is modified in the ablation experiments.

As shown in Table 4, the proposed physics-conditioned DDPM achieves the lowest $D_{\mathrm{feat}}$ value of 29.1, which is reduced by 44.8% compared with the conventional DDPM (52.7). When the impact timing or peak amplitude condition is removed, $D_{\mathrm{feat}}$ increases to 44.8 and 46.2, respectively. Since the network architecture remains unchanged across the ablation configurations, these results reflect the contributions of the physical descriptors under a fixed network architecture. The independent contribution of the cross-attention module is not quantified in the present study.

When a 10% random perturbation is introduced into the physics-conditioned information, $D_{\mathrm{feat}}$ increases from 29.1 to 35.5. Although the distribution discrepancy increases, the perturbed conditions still yield a lower distance than the conventional DDPM (52.7), indicating a degree of robustness to the simulated errors in the extracted physical descriptors.

#### 4.3.4. Experimental Validation

**Figure 13:**
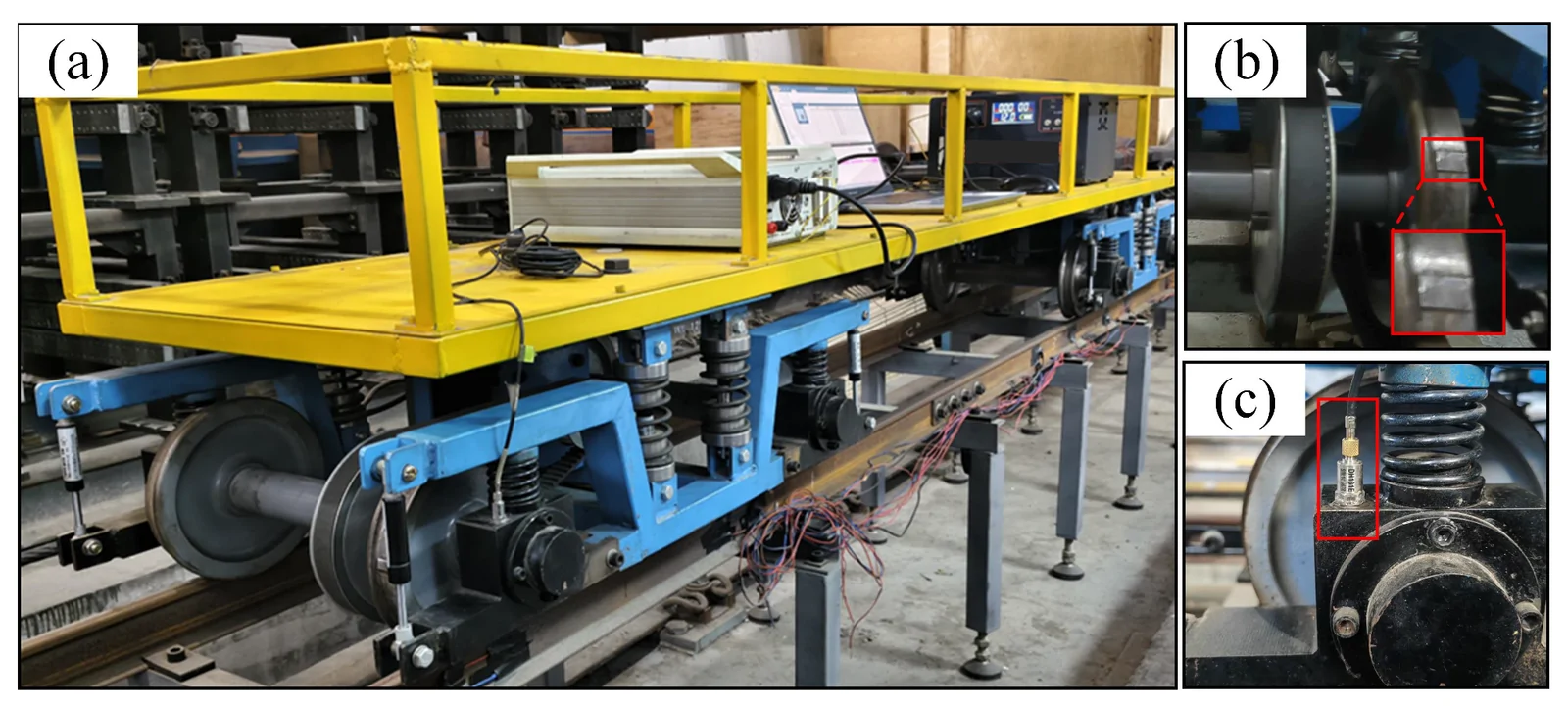
Scaled vehicle-track test rig: (**a**) vehicle, (**b**) wheel flat, (**c**) acceleration sensor.

To further evaluate the applicability of the proposed physics-conditioned DDPM under measured vibration conditions, wheel flat recognition experiments are conducted using a 1:5 scaled vehicle-track test rig. The test rig is used to reproduce wheel-rail impact excitation and vibration transmission characteristics for experimental evaluation. In the experiment, the maximum vehicle speed is 12 km/h. A wheel flat defect is introduced on the left front wheel. Acceleration sensors are employed to record the vertical axle-box vibration signals under varying operating conditions. A schematic of the scaled vehicle-track test rig is shown in Figure 13.

**Figure 14:**
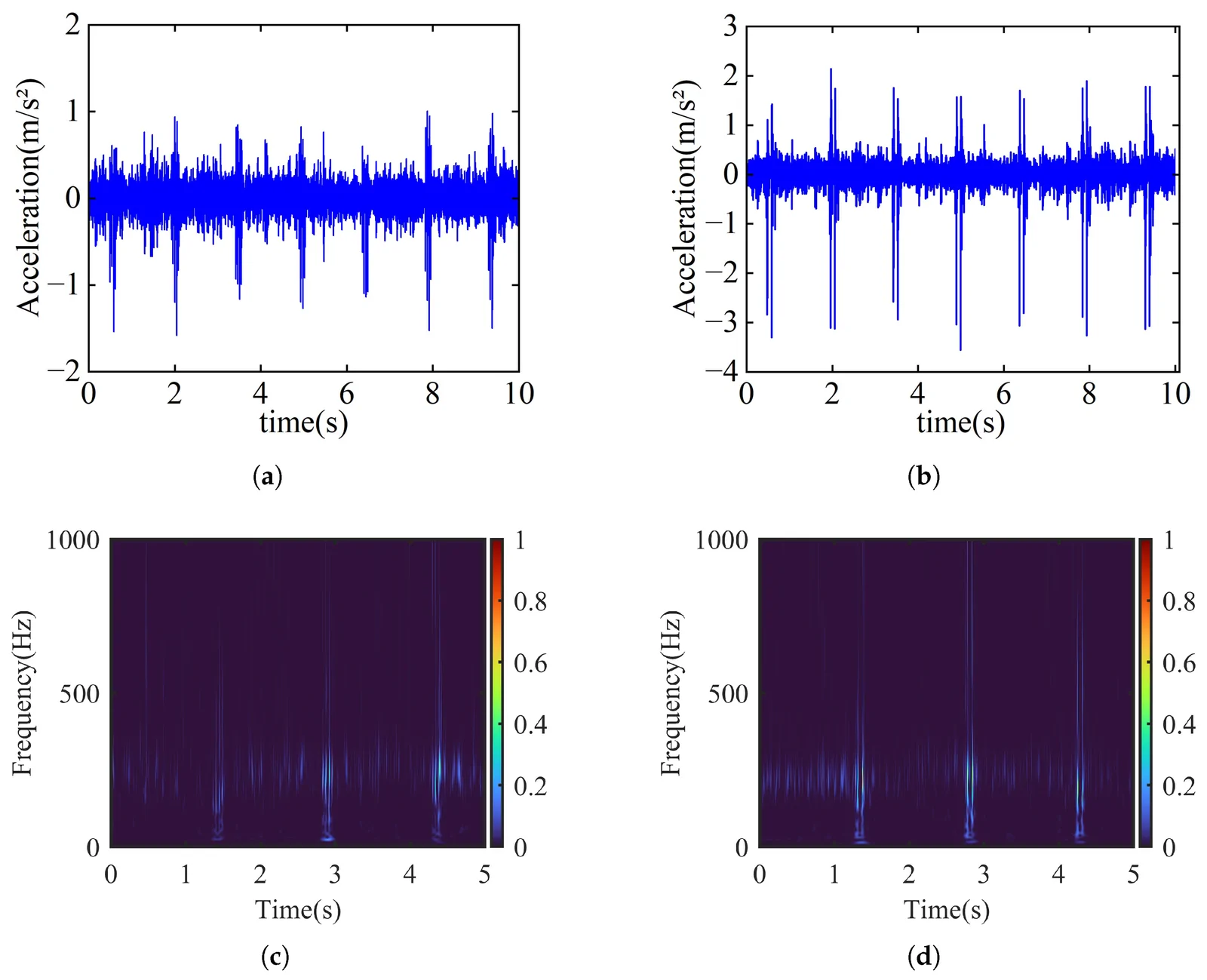
Acceleration response of the test rig: (**a**) low speed, (**b**) high speed, (**c**) low speed time-frequency diagram, (**d**) high speed time-frequency diagram.

The acceleration sensor operates at a sampling frequency of 5 kHz. During preprocessing, rail-joint-induced responses are removed according to the known spatial positions of rail joints before sliding-window sampling. Vibration segments containing rail-joint impacts are excluded to avoid interference from non-wheel-flat responses. Speed fluctuations occur during test-rig operation, and vibration responses under different defect conditions vary with operating speed and exhibit time-varying and non-stationary behavior. Partial axle-box acceleration signals are shown in Figure 14.

The five diagnostic conditions, including the no-damage condition and four wheel-flat conditions, were tested separately, and continuous axle-box vibration signals were collected for each condition. For each diagnostic condition, the recorded signal was independently divided into non-overlapping continuous segments and allocated to the training and test sets at a ratio of 2:1. Sliding-window sampling was subsequently performed separately within the training and test subsets for each condition to prevent overlapping windows from crossing the two subsets. All data augmentation operations were restricted to the training set, while the test set remained unchanged during sample generation and classifier evaluation.

A total of 750 samples were retained, with 150 samples per class and a training-to-test ratio of 2:1. Representative time-frequency diagrams are shown in Figure 14c,d. The ST-based time-frequency characteristics are consistent with those obtained from the dynamic simulations.

**Figure 15:**
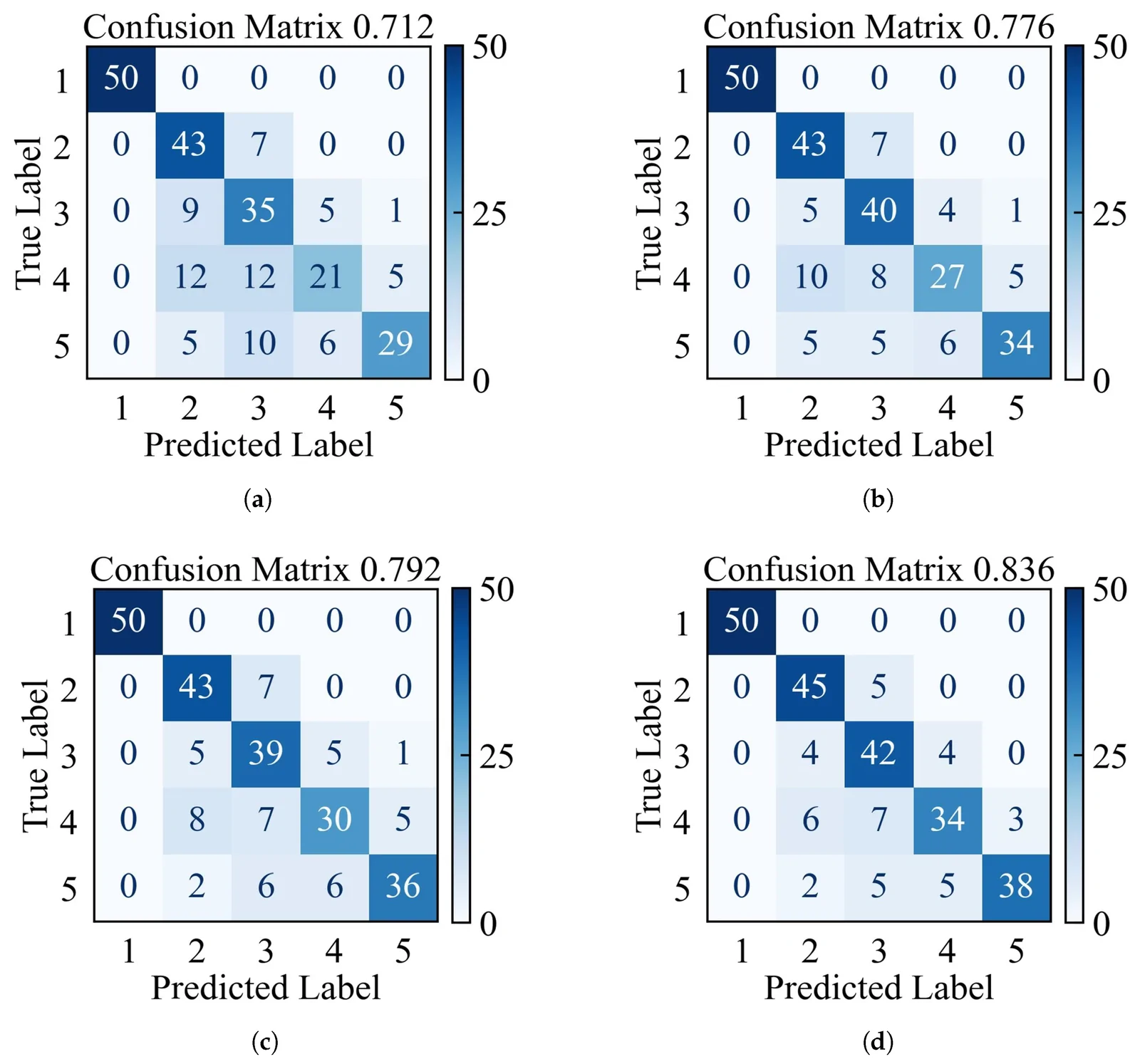
Representative confusion matrices selected from repeated training experiments: (**a**) original dataset, (**b**) DCGAN, (**c**) DDPM, and (**d**) physics-conditioned DDPM.

**Figure 16:**
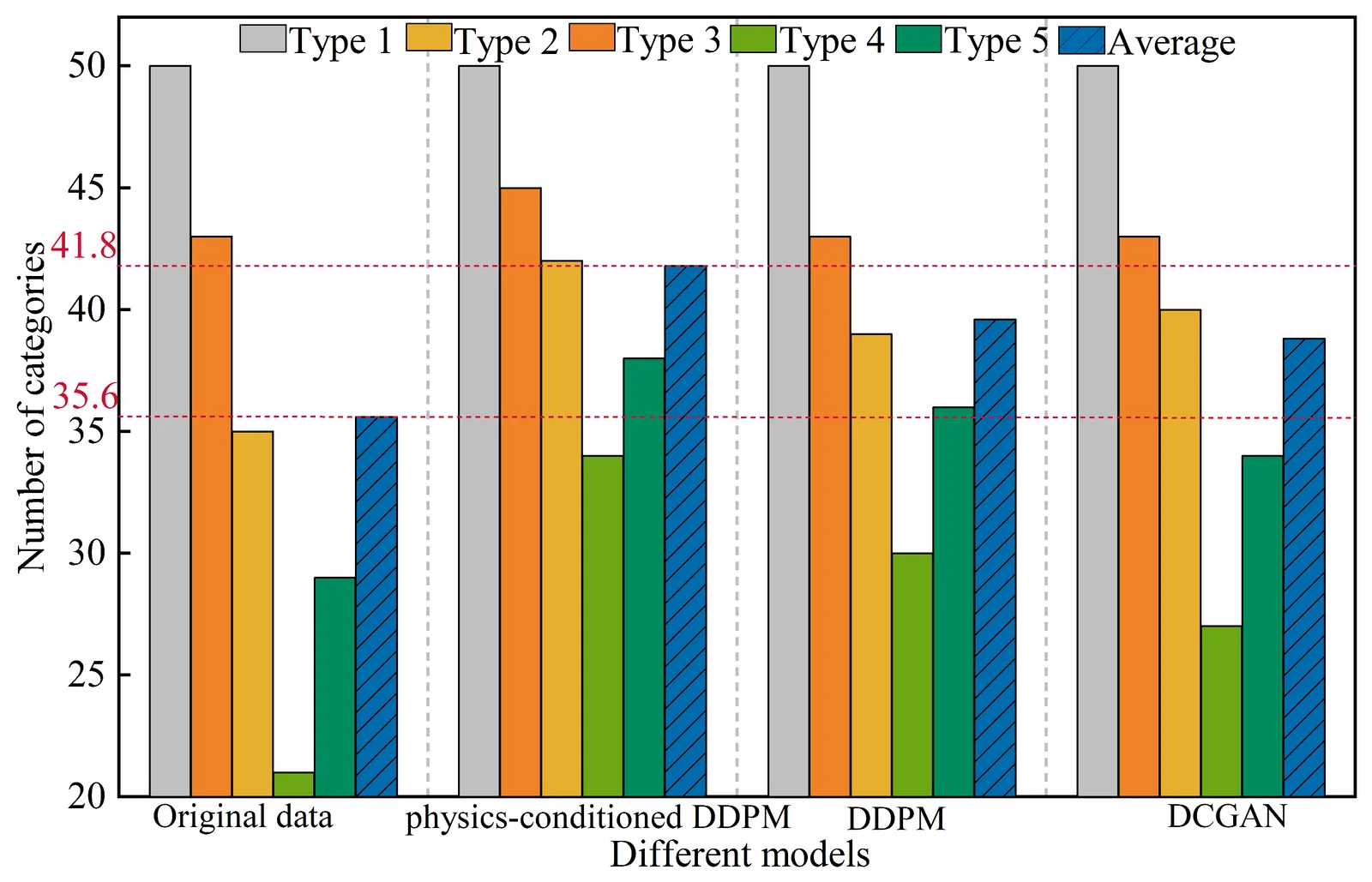
Number of correctly identified samples.

Under the same data augmentation conditions, DCGAN, DDPM, and the physics-conditioned DDPM are used for data augmentation. For each method, 100 samples are generated for each class. For the physics-conditioned DDPM, samples are generated separately for each class using the physical conditions extracted from the corresponding training samples. The generated samples inherit the corresponding class labels, and 500 generated samples are added only to the training set. To reduce the influence of random initialization, ResNet-18 is trained five times with different initializations under the same train/test split for each augmentation setting. The average accuracy obtained from the five repeated experiments is reported in Table 5. The confusion matrix corresponding to the run with the median accuracy among the five repetitions is presented in Figure 15. Based on the selected representative confusion matrices, the numbers of correctly identified samples for different classes are further summarized in Figure 16.

As shown in Table 5, under the same classifier training configuration, the dataset augmented by the physics-conditioned DDPM achieves the highest average accuracy of 81.2%. This accuracy is improved by 2.0 percentage points compared with DDPM and by 2.8 percentage points compared with DCGAN. As shown in Figure 16, compared with other augmentation methods, the physics-conditioned DDPM achieves more correctly identified samples for several wheel flat categories, especially in classes 2 and 5, where the number of correctly identified samples increases. This improvement indicates that the proposed method can generate samples with more consistent fault-related time-frequency characteristics, which contributes to improved classification performance for different wheel flat categories under measured vibration conditions. It should be noted that the scaled vehicle-track test rig provides a controlled environment for evaluating wheel-rail impact responses, but its dynamic characteristics cannot fully represent those of full-scale railway vehicles. Therefore, further validation using full-scale measurements is required to evaluate the applicability of the proposed method under practical operating conditions.

## 5. Conclusions

A physics-conditioned DDPM-based data augmentation framework is proposed for wheel flat diagnosis under variable-speed conditions. The proposed method is validated using a dynamic simulation dataset and a scaled vehicle-track test rig dataset. The main conclusions are as follows:

1. Physics-conditioned information, including impact occurrence timing and peak amplitude extracted from vehicle vibration signals, is incorporated into the diffusion generation process through two-dimensional condition maps. With the incorporation of physics-conditioned information, the DDPM can improve the preservation of impact characteristics and time-frequency evolution patterns of wheel flat faults under variable-speed conditions. The feature distribution distance of the generated samples in the simulation dataset is reduced to 29.1, which is lower than that obtained using the conventional DDPM. This result indicates that the introduced physical conditions can effectively reduce the distribution discrepancy between generated and real samples.
2. After classifier training with augmented datasets generated by the proposed method, wheel flat diagnosis performance is improved on both datasets. For the dynamic simulation dataset, the recognition accuracy is increased from 87.8% to 94.4% compared with the original dataset. For the scaled vehicle-track test rig dataset, the recognition accuracy is improved from 69.2% to 81.2%. These results demonstrate that the proposed physics-conditioned DDPM can alleviate the problem of insufficient fault samples and improve wheel flat diagnosis performance under variable-speed conditions.

However, the proposed method is currently evaluated using predefined acceleration–deceleration speed profiles, while the experimental validation is limited to a 1:5 scaled vehicle-track test rig with a maximum speed of 12 km/h and a limited range of wheel-flat defect sizes. These conditions do not fully represent the complexity of full-scale railway operation. Since vehicle speed is not directly used as the input condition, the generalization capability of the proposed method under unseen speed trajectories and more diverse operating conditions requires further investigation. In future work, additional vehicle-track dynamic parameters, such as wheel-rail interaction characteristics and suspension responses, will be incorporated, and the generalization capability of the proposed method will be further evaluated under wider speed ranges, different defect severities, and more complex operating conditions.

## Figures and Tables

**Table 1:** Simulation parameters.

Parameter	Value
Wheel diameter *D*	885 mm
Wheel flat length *L*	20, 30, 40, and 50 mm
Vehicle speed profile	40–100 km/h (a=1 m/s^2^), 100–40 km/h (a=−1 m/s^2^)
Sampling frequency	10 kHz

**Table 2:** Physics-conditioned DDPM augmentation performance.

	750 Generated Samples	1500 Generated Samples	3000 Generated Samples
Accuracy	93.2%	94.4%	92.6%

**Table 3:** Augmentation results of different generative models.

	Ratio of 0.5	Ratio of 1
Accuracy (DCGAN)	90.2%	91.0%
Accuracy (DDPM)	90.6%	91.2%
Accuracy (Physics-conditioned DDPM)	93.2%	94.4%

**Table 4:** Ablation study and robustness analysis.

Method	$D_{\mathrm{feat}}$
DDPM	52.7
without impact timing	44.8
without peak amplitude	46.2
with 10% condition perturbation	35.5
Physics-conditioned DDPM	29.1

**Table 5:** Test accuracy comparison of different augmentation methods.

	Original Dataset	DCGAN	DDPM	Physics-Conditioned DDPM
Accuracy	69.2%	78.4%	79.2%	81.2%

## Data Availability

The datasets generated and analyzed during the current study are available from the corresponding author upon reasonable request due to restrictions related to the research project.
